# Puffy Skin Disease Is an Emerging Transmissible Condition in Rainbow Trout *Oncorhynchus mykiss* Walbaum

**DOI:** 10.1371/journal.pone.0158151

**Published:** 2016-07-08

**Authors:** Irene Cano, David W. Verner-Jeffreys, Ronny van Aerle, Richard K. Paley, Edmund J. Peeler, Matthew Green, Georgina S. E. Rimmer, Jacqueline Savage, Claire L. Joiner, Amanda E. Bayley, Jason Mewett, Jonathan Hulland, Stephen W. Feist

**Affiliations:** Aquatic Animal Disease, Centre for Environment, Fisheries and Aquaculture Science, Barrack Road, The Nothe, Weymouth, Dorset DT4 8UB, United Kingdom; Friedrich-Loeffler.Institut, GERMANY

## Abstract

The transmission of puffy skin disease (PSD) to rainbow trout *Oncorhynchus mykiss* Walbaum was tested in the laboratory by conducting co-habitation challenges with puffy skin (PS)-affected fish (Trojans) collected from the field. Two separate challenges were conducted using Trojans sourced from two different sites and diploid (first trial) or triploid (second trial) naïve fish. PSD-specific clinical signs were observed in both groups of naïve fish, with 66% of the fish sampled during the challenges showing signs of varying severity. The first clinical features of PSD were presented as white oval skin patches on one or both flanks 15–21 days post-challenge (dpc). The extent of the lesions ranged from 10 to 90% of the body surface, depending on the severity of the lesion. Both the severity and number of affected fish increased during the challenge. Macroscopically, oedema of the skin and multifocal petechial haemorrhaging were observed towards the end of the trials. Abnormal fish behaviour consisting of “flashing” and excessive mucous production was noted from 15 dpc onwards. Fish with severe PSD lesions also displayed inappetence and associated emaciation. Rodlet cells were observed in 41% of the fresh skin scrapes analysed from the second trial. Histologically epidermal oedema was observed in 31% of the naive fish showing gross pathology, with additional 12% displaying epidermal hyperplasia, mostly observed at the end of the challenge. Other concomitant features of the PSD lesions in challenged fish were epithelial erosion and sloughing, and occasionally mild or focal inflammation. No consistent pathology of internal organs was observed. The parasites *Ichthyophthirius multifiliis* and *Ichthyobodo necator* were observed in skin samples of a proportion of naïve challenged fish and in Trojans but not in control fish. The presence of these and other known fish pathogens in the skin of PSD-fish was confirmed by high-throughput sequencing analysis. In summary, we have demonstrated that PSD is a transmissible condition. However, even though a number of known fish pathogens were identified in the skin tissues of PSD-fish, the actual causative infectious agent(s) remain(s) unknown.

## Introduction

In 2002 a new skin condition in farmed rainbow trout *Oncorhynchus mykiss* (Walbaum) in England was first recognised by the Cefas Fish Health Inspectorate (FHI), and became known as puffy skin disease (PSD) [1]. The condition persisted on affected farms but the apparent rate of spread to new sites was low until 2006, when reported cases increased substantially [1]. From 2012 onwards, PSD has been also reported in rainbow trout fisheries [2]. In those fisheries affected, as well as in fish farms, the most severe PSD lesions and highest number of fish affected were observed in late summer and autumn. PSD was typically only observed in rainbow trout, and not in brown trout *Salmo trutta* (L.) in the same ponds (FHI unpublished observations).

PSD was first formally reported as an emerging skin disease for rainbow trout as part of a study that characterised a variety of skin conditions of uncertain aetiology observed in rainbow trout [3]. Subsequently, a more detailed case definition for PSD was established [2]. In the water, affected fish appear grey in colour and on closer inspection affected areas of skin, normally on the flank, show excessive mucous and dermal opacity. In advanced cases, a thick mucus layer covers areas of raised scales and skin. Affected fish may lose appetite and become emaciated. The condition does not usually result in mortality but can lead to economic loss due to increased production costs, culling and downgrading of carcasses at slaughter.

An epidemiological review, based on the results of a questionnaire survey of English and Welsh rainbow trout farms, reported PSD on 37% (n = 49) of rainbow trout sites, located in 28 river catchments across England and Wales. That study highlighted that sites receiving live rainbow trout in the last 12 months were considerably more likely to have PSD, also suggesting an infectious aetiology [1].

An infectious agent has been suggested for another important skin condition that affects rainbow trout in the US and Europe, namely cold water strawberry disease (CWSD) or red mark syndrome (RMS) after field investigations and controlled transmission trials [3,4]. A definitive causative agent is still to be identified, although an association with a rickettsia-like agent (RLO) has been suggested [5–7].

The aim of this study was to further test the hypothesis that PSD has an infectious aetiology, by undertaking a cohabitation experiment whereby naive triploid and diploid rainbow trout were cohabited with PSD-fish collected from the field. In addition, extensive additional investigations, including analysis of high-throughput sequencing datasets generated from skin samples of PSD-fish were undertaken in an attempt to identify potential infectious agents that may be responsible for PSD.

## Materials and Methods

### Co-habitation challenge design

Two challenge trials based on cohabitation of naïve rainbow trout with PSD-fish were carried out (Table 1). In order to determine if the condition can be transmitted to triploid rainbow trout, as observed in the field, but also to diploids, different stocks of naïve fish were used in each challenge.

**Table 1:** Experimental design of transmission experiments. For both experiments, fish were held at 15°C for 49 days after initiation of the cohabitation challenge in 300 L tanks. In all cases, the naïve fish used were marked with elastomer tags prior to the start of the experiment. With the exception of the naive fish used for Trial 1, all fish used were triploid stocks.

Trial	Time	Source of naive fish	Source of cohabitant (Trojan) fish	Experimental design summary	Sampling
1	Winter 2013	Fish reared in house from eggs (diploid)	Site A	15 fish from site A mixed with 20 naïve fish in a single tank	Trojan fish sampled prior to start of challenge. Naïve fish sampled at 19, 23, 28 and 41 dpc
2	Summer 2014	Site C. No history of PSD on farm. Fish held 12 days at CWL prior to start of experiment	Sites A+B	Tank 1: 39 naïve fish from site C mixed with 15 fish from Site A	Trojan fish sampled prior to start of challenge. Naïve fish sampled at 14, 21, 35 and 49 dpc
				Tank 2: 39 naïve fish from site C mixed with 15 fish from Site B	
				Tank 3: negative control group. Fish from site C held for the duration of the experiment	

#### Experimental fish

Rainbow trout showing clinical signs of PSD (ca. 450g in weight) were provided by two farms (Sites A and B) and transported to the Cefas Weymouth Laboratory (CWL) to be used in the two transmission challenges in this study. The PSD-affected farms (Site A and Site B) were from the same catchment (a spate river in the South West of England). One unit was a small scale enterprise (5–10 tonnes production per annum) with a mixture of approximately 10 small (approx 15m^2^) earth ponds and concrete tanks. Site B was larger scale (50–100 tonnes per annum production) comprising approximately 20 larger (approx. 100m^2^) earth ponds.

Fish on both sites were raised from imported fry until market size (>400 g) and had a history of recurrent PSD outbreaks. Both sites were supplied with water from and discharged to adjacent river courses. They both sourced fry from different suppliers. They had both reportedly shared the same suppliers on occasions and, although they were based in the same water catchment, they did not use the same water course.

The "PSD negative" site Farm C was a closed site (no live fish movements onto the site with fish reared from hatched eggs) in the north of England producing approximately 100 to 200 tonnes of rainbow trout per year in approximately fifty 50–100 m^2^ earth ponds. It had no history of PSD or RMS and there was no movement of live fish onto the site as it has its own hatchery and broodstock. As with the other two sites, it took water from a nearby river.

Naïve fish used in the experiments were sourced either from in-house reared fish from eggs (first challenge trial) or from a third site (Site C; second challenge trial).

The freshwater used for the challenges was derived from chalk and limestone boreholes 10 km from the laboratory, dechlorinated at 10 ± 0.5°C and supplied to all tanks at a rate of 1–3 L min^–1^ from the laboratory’s potable water supply. All tanks were maintained as separate flow-to-waste systems (with effluent water treated by ozonation prior to discharge). As part of CWL-standardised procedures, strict biosecurity was implemented to minimise the risks of transfer of potentially infectious material among tanks.

First challenge trial (Trial 1). PSD-fish collected from an affected fish farm (Site A, December of 2013) were acclimated to 15°C in tanks at CWL and held for 4 days before the cohabitation challenge. Before the challenges were initiated, a sub-sample of ten fish was assessed histologically for the severity of PSD and background disease status. Fish were euthanised and samples taken for bacteriological, virological and parasitological examinations, as described for the fish sampled from the trials.

Adult female diploid rainbow trout, reared in-house, weighing 250–400 g, were tagged 4 days prior to challenge using visible implant elastomer (VIE) tags (Northwest Marine Technology, USA) following the manufacturers’ instructions. All fish were fasted for 24 h prior to tagging then lightly anaesthetised in MS222 (Sigma, UK). Fish were individually taken to a clean work table, where they were injected with elastomer underneath the area of non-pigmented skin immediately posterior to the eye. Tagged fish were then returned to their original tank and observed to confirm that they had safely recovered from anaesthesia. Supply of feed was resumed 24 h post-tagging. Marked fish were then identified by visual observation of the tags by using a handheld deep violet light.

On the challenge day, cohabitation was initiated by exposing 20 tagged naïve fish to 15 PSD-affected rainbow trout. Fish were observed daily for up to seven weeks for signs of infection. Any relevant observations were noted, including changes in behaviour and/or appetite and clinical signs.

Second challenge trial (Trial 2). In the second challenge, PSD-affected ‘Trojan fish’ from two different geographical sites, site A and site B, were collected in July of 2014, acclimated to 15°C and held for 6 days before the cohabitation challenge. A health assessment of these fish was undertaken as for Trial 1.

On this occasion, naïve triploid fish (weighing 200–300 g) collected from a fish farm without any record of PSD were held in two 1000l tanks, each holding 39 fish. These fish were held for 12 days prior to the start of the experiment. The fish were VIE tagged (as described above for Trial 1) and exposed to PSD by cohabitation with 15 PSD-fish from each site in a separate tank. In parallel, 54 VIE tagged naïve fish were held in a third tank as negative controls.

### Observations and sampling

For both trials, fish were observed daily for up to 7 weeks for signs of infection. Observations, including changes in behaviour and/or appetite and clinical signs were noted. A severity index to rank the gross pathology of the lesions that developed in challenged naïve fish was applied following the criteria developed by Maddocks et al. [2].

For the first trial, fish were sampled at 19, 23, 28 and 41 days post-challenge (dpc). A ratio of naïve-Trojan fish in the tank of approximately 4:3 was maintained until the end of the challenge. For the second trial, samples were taken at 14, 21, 35 and 49 dpc with a naïve-Trojan ratio of 3:1. Tissue samples of skin, gills and internal organs were taken and processed for histology, microbiology, virology and sequencing as described below.

### Skin scrapes and histology

Mucous samples obtained from skin smears were observed in the fresh state under a light microscope using Nomarski interference contrast. Some of the skin smears were air dried and stained with Giemsa stain.

Tissue sections of skin, gills, heart, brain, liver, kidney, spleen and intestine of each sampled rainbow trout were fixed in 10% neutral-buffered formalin (NBF) for 24 h. Tissues were embedded in paraffin wax using a vacuum infiltration processor (Leica Biosystems) following standard protocols. Embedded blocks were sectioned at 3−4 μm thickness using a rotary microtome (Shandon Finesse) and sections were stained with hematoxylin and eosin. Sections were examined using a Nikon E800 light microscope with images captured using Lucia^™^ software. The tissues sections were examined blind and the presence of lesions recorded.

### Bacteriology examinations

Skin and head kidney swabs were plated onto tryptic soy agar (TSA)[8], Mueller-Hinton agar[9] (MHA)[10], marine agar (MAR)[11], Anacker and Ordal’s agar (AOA) [12], and tryptone yeast extract salts (TYES) [13] agar. Plates were then incubated at 15°C and observed daily. Discrete colonies of interest from a mixed population were isolated and subjected to Gram stain, catalase, oxidase, oxideation/fermentation and motility tests following standard protocols[14].

For some of the isolated colonies the bacterial DNA was extracted using an EZ1 DNA mini Kit and EZ1 extraction robot (Qiagen, Manchester, UK), eluting the DNA in 60 μL of elution buffer. The 16S rRNA gene of some of the isolated colonies was PCR amplified using the universal primers previously described [15], giving a fragment of 600 bp. PCRs were performed in a 50 μL reaction volume consisting of 1x GoTaq flexi buffer (Promega, UK), 2.5 mM MgCl_2_, 1 mM dNTP mix, 50 pmol of the forward and reverse primers, 1.25 units of GoTaq^®^ DNA Polymerase (Promega, UK) and 2.5 μL of the purified DNA. The reaction mix was overlaid with mineral oil and after an initial denaturing step (5 min at 95°C), was subjected to 35 temperature cycles (1 min at 95°C, 1 min at 55°C and 1 min at 72°C) in a PTC-225 Peltier thermal cycler (MJ Research, Canada) followed by a final extension step of 10 min at 72°C. PCR products were visualised on 2% agarose gels stained with ethidium bromide. Direct sequencing analysis of the PCR products were performed using the Applied Biosystems 3130/3131x/Genetic Analysers, Big Dye Terminator V1.1 Cycle Sequencing Kit (Applied Biosystems). Editing and assembly of sequence trace (ABI) files and alignments of contigs were performed using the Sequencer program from Gene Codes Corporation.

### Virology analysis

From each sampled fish a skin section, and a pool of head kidney, spleen, heart and brain were homogenized 1:10 in L-15 media supplemented with 1 mM L-glutamine, 10% fetal bovine serum, 1% antibiotic-antimycotic solution (Gibco, Life Technologies, Paisley, GB), 5% glutamax, 0.16% Trizma base solution (Sigma, Poole, GB) and 0.48% sodium bicarbonate. clarified by centrifugation for 10 min at 2500g and inoculated onto four different fish cell lines: chinook salmon embryo (CHSE-214) (ATCC^®^: CRL-2872^™^); a salmonid cell line derived from head kidney (TO) [16]; epithelioma papulosum cyprini (EPC) (ATCC^®^: CRL-2872^™^); and bluegill *Lepomis macrochirus* fry (BF-2) (ATCC^®^: CCL-91^™^; [17]).

Prior to inoculating the cells, a fraction of each sample was pre-treated with infectious pancreatic necrosis virus(IPNV) neutralizing antisera (Polyclonal goat anti-Serotype Sp IPNV serum; Harlan Sera-lab). Then, each sample, with and without IPNV antisera, was inoculated at 1/10 and 1/100 and incubated at 15°C for 7 days with regular observation for development of cytopathic effects (CPE). Monolayers were blind passaged onto fresh cells and observed for a further seven days.

Samples developing CPE were subjected to an IPNV Ag ELISA test (TestLine Clinical Diagnostics, Czech Republic).

### Metagenomic sequencing

Tissue samples were collected from two naïve fish showing PSD clinical signs after cohabitation with PSD-fish. For each fish, skin and muscle sections were taken from both affected and non-affected skin regions (samples 11-A and 15-A and 11-N and 15-N, respectively). RNA samples were prepared as described previously [18] and double-stranded cDNA was generated with random primers using Sequenase (Affymetrix, UK). DNA sequencing libraries were prepared using Nextera XT kits (Illumina, UK) and sequenced on an Illumina MiSeq (using the 2x150bp paired-end protocol) at the Animal Health and Veterinary Laboratory Agency (AHVLA, UK). All raw sequence data are accessible at the NCBI Sequence Read Archive through accession number SRP076264.

Adapter sequences and low quality bases were removed using Trimmomatic (v0.32 [19]). A number of different approaches were used to identify potential pathogen sequences in the dataset. Firstly, all of the paired, quality-trimmed reads were screened for the presence of small (16S/18S) and large (23S/28S) subunit rRNA genes using Metaxa2 [20]. Family-level taxonomic counts were generated, normalized according to the number of reads in each sample and compared across the 4 samples using R/Bioconductor [21]; following the method described on: http://metagenomics-workshop.readthedocs.org/en/2014-5/annotation/metaxa2.html. Secondly, gene transcripts were assembled *de novo* for each sample using the Trinity assembly pipeline (r20140717; [22]) and default parameters. Prior to assembly, reads representing rainbow trout sequences were removed by discarding reads that mapped to the full set of rainbow trout nucleotide sequences in the NCBI nucleotide (nt) database (14/09/2014) using bowtie [23] and paired up using a modified python script (see Supplemental Information for more details). Resulting transcripts were annotated using Blastn [24] and the full NCBI nucleotide (nt) database (14/09/2015).

## Results

### PSD-affected and naïve fish used in challenge trials

No viral or bacterial pathogens were detected in the naïve diploid rainbow trout that were reared at Cefas and used for the first challenge trial. However, in the second batch of naïve fish (triploid rainbow trout obtained from the field), IPNV was detected in 1 out of 10 fish sampled, with a titre ranging from 10−10^2^ TCID_50_ mL^-1^ (Table 2). IPNV-positive fish did not show gross pathology or histopathological changes when examined (Table 2).

**Table 2:** Background disease status of naïve and PSD affected (Trojan) fish used in cohabitation experiments. Severity of puffy skin lesions rated as mild, moderate and severe. Number of positive fish given in percentage (n = 10 per group).

Trial	Fish group	Puffy Skin	Bacteriology	Virology	Parasites
1	Trojan (Site A)	20% mild to moderate	*Shewanella*-like (2/10)	-ve	-ve
	Naïve (in house reared)	-ve	-ve	-ve	-ve
2	Trojan (Site A)	60% mild to moderate	*Shewanella*-like (3/10)	-ve	-ve
	Trojan (Site B)	60% severe, 40% moderate	*Aeromonas salmonicida* (1/10)	-ve	-ve
	Naïve (Site C)	-ve	-ve	IPNV (1/10)	-ve

Trojans from site A, consisting of fish displaying PSD clinical signs, were collected twice, in winter (December) for the first trial, and in the summer (June) for the second trial. In the group collected in winter, 20% of analysed fish presented with mild to moderate PS clinical signs, but no recognised pathogens were detected in the samples analysed. In the batch of fish collected in the early summer, that presented with mild to moderate PS clinical signs in 60% of the fish analysed, phenotypically *Shewanella*-like isolates (Gram negative, oxidase and catalase positive, oxidation /fermentation test negative), and forming distinctive pinkish tinged colonies when incubated on TSA), were isolated from 30% of the fish analysed from skin and head kidney swabs. The partial 16S rRNA gene from one of these isolates was amplified and partially sequenced. Comparison with sequences derived from Type strains using the tool Seqmatch [25] confirmed this was likely to be a member of the Aeromonadaceae with closest, but still limited, similarity to *Shewanella* spp. (closest S_AB score of 0.816 to AF005249 derived from *Shewanella algae* Type Strain ATCC 5119)

For the fish sourced from site B, 60% of the fish analysed in the health check displayed severe skin lesions characteristic of puffy skin condition (white patches and oedematous changes on the skin) (Table 2). Apart from PSD, skin lesions, or ‘furuncles’, characteristic of furunculosis [26], were observed in less than 5% of fish from Site B used in the second experiment upon arrival, and confirmed later with the isolation of *Aeromonas salmonicida* from these fish. Any fish showing clinical signs of furunculosis were removed from the PSD transmission experiments during the quarantine and acclimation phase.

### Transmission of puffy skin condition

#### Trial 1

As a proof of concept a single tank containing diploid rainbow trout was challenged with PSD-fish (Table 1). After 15 dpc naive fish were observed to show irregular swimming, or ‘flashing’, with fish swimming down and rubbing their flanks on the bottom of the tank, then rapidly righting themselves. This behaviour was observed intermittently during the challenge in different fish. Excess mucous production was evident by the amount of increased mucous levels observed on the water surface from 15 dpc onwards.

After 15–21 dpc, clinical signs of PSD presented as white oval patches in the skin of one or both flanks coinciding with irregular swimming behaviour and excessive mucous production. Patches were first observed around the lateral line, and the extension of the patches ranged from 10 to 90% of the skin, depending on the severity of the lesion together with raised scales. Scale loss was commonly observed in the sampled fish. Inappetence was also recorded in affected fish. Fish sampled at 19 dpc did not show pathological changes, despite the excessive production of mucus (Table 3).

**Table 3:** Clinical signs, microbiology and histopathology findings of naïve fish sampled during the first challenge trial after exposure to cohabitant PSD Trojan. In parenthesis: number of positive fish/total fish sampled. NSO: nothing significant observed; NAD: no abnormalities detected; HK: head kidney.

	19 dpc	23 dpc	28 dpc	41 dpc
*No*. *fish sampled*	3	3	4	10
*Behavioural signs*	Flashing	Flashing	Flashing	Flashing
	General lack of appetite	General lack of appetite	General lack of appetite	General lack of appetite
*Gross clinical signs*	Severe skin mucus production (3/3)	White patches affecting 25–80% of skin surface (1/3)	White patches affecting 60% of skin surface (4/4)	Dermal hyperplasia, skin petechial haemorrhages (4/10)
	Scales raised and loss (3/3)	Epidermal oedema (1/3)		
*Virology*	-ve	-ve	-ve	-ve
*Bacteriology*	Skin: *Acidovorax* sp. (1/3)	Skin: *Acidovorax* sp. (3/3)	Skin: *Acidovorax* sp. (1/4)	Skin: *Acidovorax* sp. (10/10)
	HK: NSO	HK: Acidovorax sp. (1/3)	HK: NSO	HK: Acidovorax sp. (2/10)
*Skin histopathology*	NAD	Raised scales (2/3)	Raised scales (4/4)	Raised scales (1/10)
		Epidermis oedema, dermatitis (1/3)	Epidermis oedema, dermatitis (1/4)	Scales pocket oedema (8/10)
		Epithelial erosion, sloughing (2/3)	Epithelial erosion, sloughing (4/4)	Epidermis oedema, dermatitis (2/10)
		Inflammation (1/3)		Dermal hyperplasia (2/10)
*Internal organ histopathology*	NAD	NAD	NAD	Epicarditis (3/10)
				Renal Haemorrhaging and focal necrosis (1/10)
				Spleen focal haemorrhaging, inflammation (2/10)
*Parasites*	-ve	*Icththyophthirius multifiliis* (2/3)	-ve	-ve

**Fig 1:**
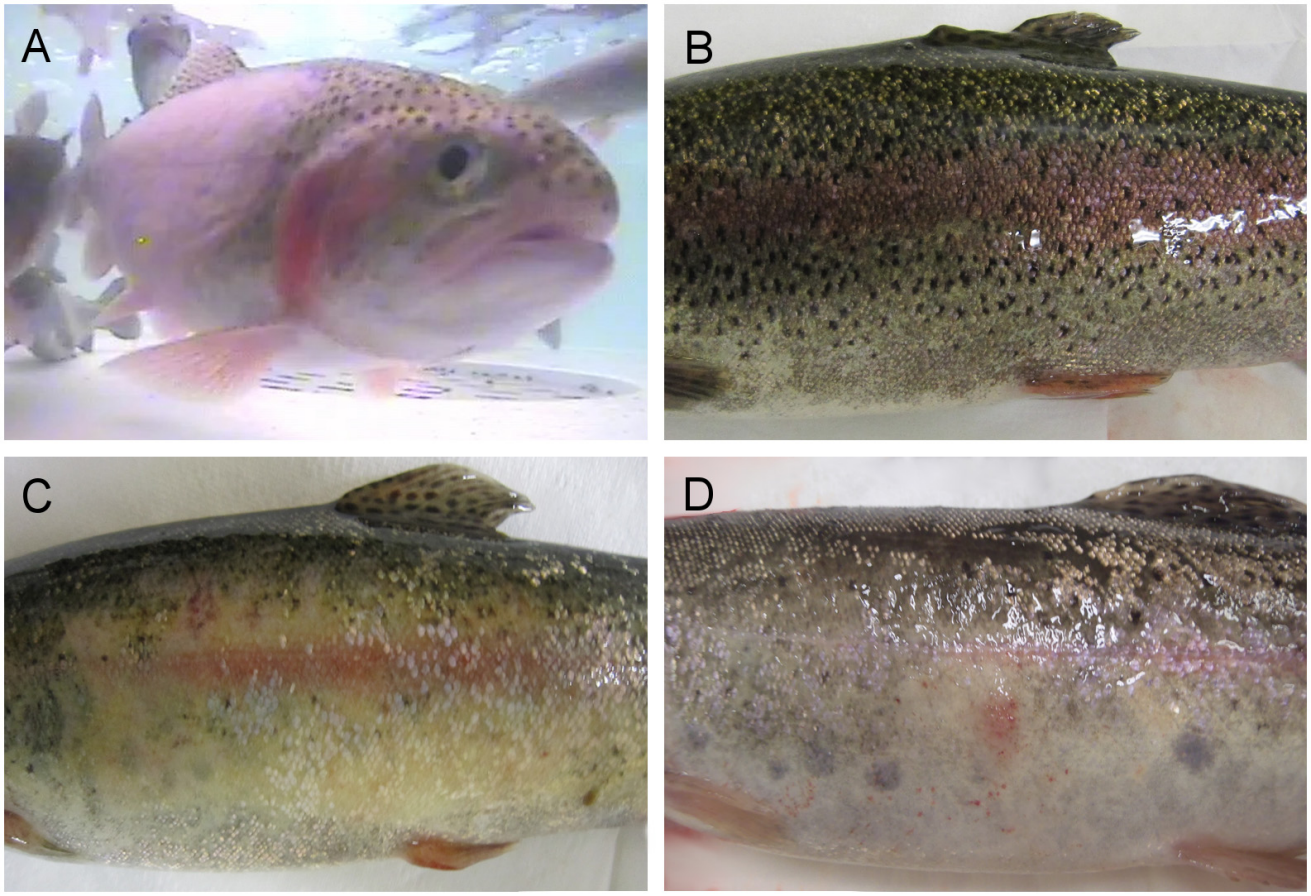
Puffy skin disease (PSD) in naïve rainbow trout after cohabitation with PS-affected fish. (A) Naïve fish in a cohabitation tank showing oedematous changes in the skin. (B) Control fish. (C) Naïve fish showing an oval white patch affecting 80% of one flank and excessive mucus production. (D) Multifocal epidermal hyperplasia and petechial haemorrhaging distributed over the affected area.

However, at 23 dpc, macroscopic epidermal oedema, characteristic of PSD lesions, was observed in one out of three naïve fish sampled, with 25 and 80% (each flank respectively) of the skin affected. At the end of the challenge (Day 41; Table 3), four out of ten fish showed macroscopic epidermal hyperplasia. However, of these in only two of the fish were PSD lesions confirmed histologically. Those fish with severe PSD presented multifocal petechial haemorrhaging (Fig 1). Other histopathological changes observed in the skin were raised scales, scale pocket oedema, epithelial erosion and sloughing. The parasite *I*. *multifiliis* was identified in two out of three fish sampled 23 dpc in histology examinations. Mild epicarditis and focal renal and splenic haemorrhaging were observed at the end of the challenge in three and two out of ten fish sampled respectively, although not in fish exhibiting oedematous changes in the skin (Table 3). No parasites were observed in skin scrapes analysed throughout the challenge.

#### Trial 2

In order to test the robustness of the challenge, a second trial, including a control tank and Trojans from two different geographical sites, was carried out.

**Fig 2:**
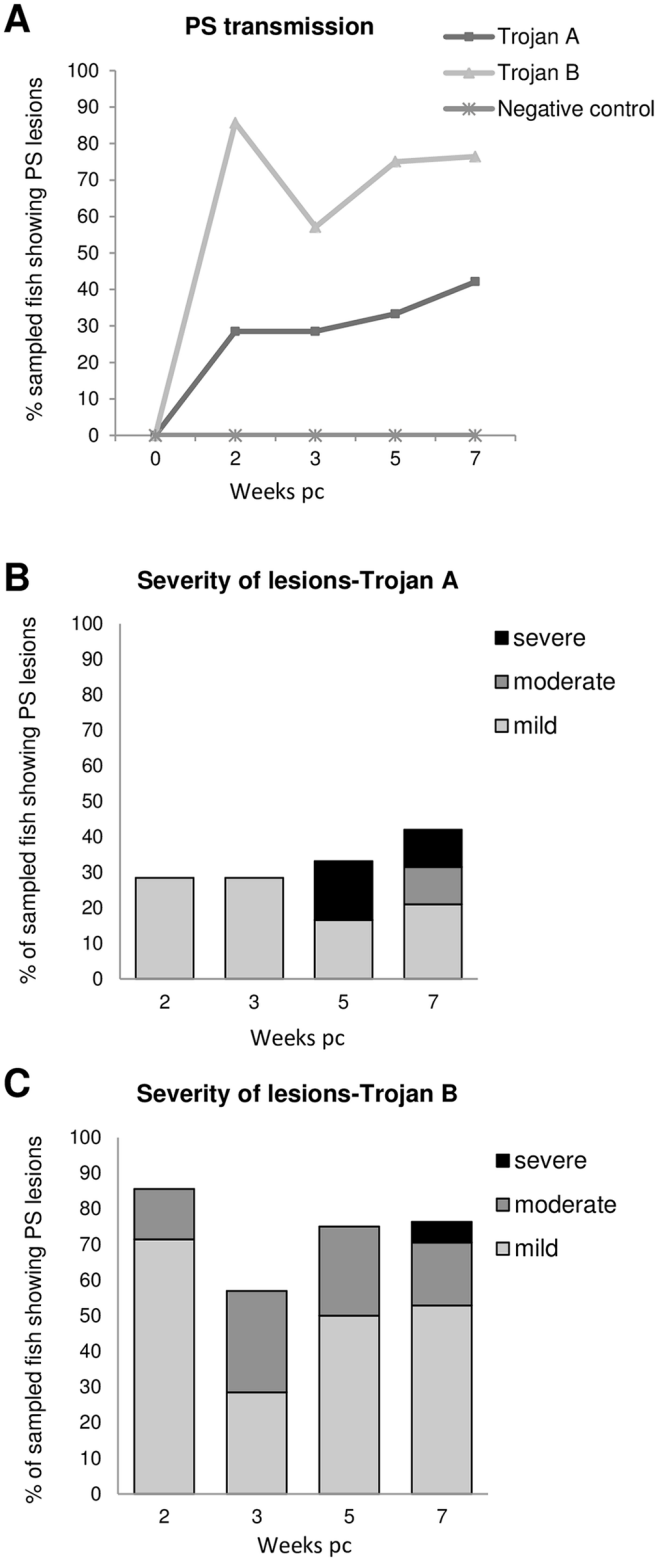
Second cohabitation challenge with PSD affected-fish (Trojans). (A) Percentage of sampled fish showing PSD clinical signs at each sampling point. (B, C) Severity of PSD lesions in naïve fish cohabitating with Trojan from site A (B) and with Trojan from site B (C). PSD severity ranking: mild: white patches in one or both flanks; Moderate: focal epidermal hyperplasia; severe: multifocal areas or epidermal hyperplasia, petechial haemorrhaging.

Trial 2 Clinical signs, gross pathology. Similar to the first trial, some naïve fish cohabited with Trojans started to display irregular swimming (flashing) after 15 dpc. PSD severity was ranked as mild, moderate or severe (Fig 2). Fish from the negative control tank showed no clinical signs or unusual behaviour.

Naïve fish cohabitated with Trojan fish from site A developed multifocal epidermal hyperplasia, ranked as severe PSD, which was most evident in fish sampled at 35 and 49 dpc. Other fish sampled showed mild lesions, with white patches on the skin from 14 dpc. At the end of the challenge 45% of the fish sampled showed skin lesions related to PSD. Inappetence was also noted during the challenge.

Naïve fish in cohabitation with Trojan fish from site B showed a higher percentage of fish with PSD lesions compared with those cohabiting with Trojan fish from Site A. At 14 dpc 85% of the fish displayed mild or moderate PSD lesions. At the end of the challenge, 80% of the naïve fish were affected, although severe lesions were observed in only 5% of the fish sampled.

**Fig 3:**
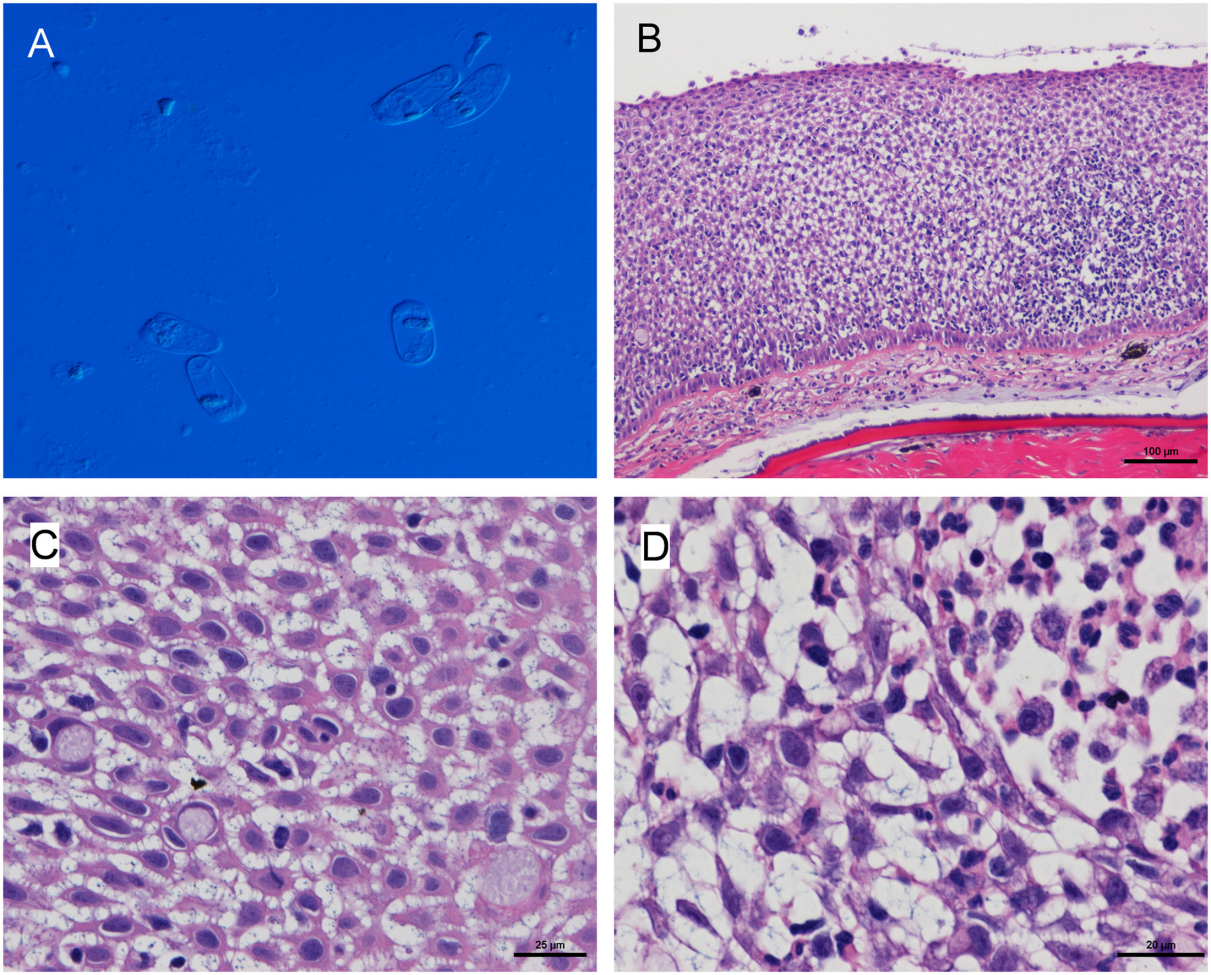
Second puffy skin disease (PSD) challenge trial. (A) Rodlet cell observed in skin scrapes from challenged naïve fish, (B, C, D) Haematoxylin & eosin stain of a skin histological section showing epidermal hyperplasia and dermatitis developed in a naïve fish in cohabitation with PSD-affected fish.

Trial 2 skin smear analysis. In the naïve cohabitant fish 69.5% of the skin smears analysed (n = 23) showed abundant rodlet cells (Fig 3A). In 35% of these cases naïve fish showed typical PSD signs, namely oedema, dermatitis and/or epithelial hyperplasia. Rodlet cells were not observed in skin smears analysed from Trojan fish (n = 15) neither in control fish (n = 3). *Ichthyobodo necator* was identified in 10% of the skin smears from Trojan fish of both sites but not in challenged naïve fish.

Trial 2 Histopathology. Histological analysis of naïve fish in cohabitation with PSD-fish showed epithelial hyperplasia and oedematous change typical of PSD (Fig 3B), confirming the transmission of the PSD condition. Histopathological alterations in the skin are shown in Table 4.

**Table 4:** Histopathological changes observed in skin of rainbow trout during the second cohabitation trial with puffy skin disease affected fish.

Skin	Negative control				Naïve cohab with TA				Naïve cohab with TB				TA			TB			
dpc	0	2	3	7	2	3	5	7	2	3	5	7	0	5	7	0	3	5	7
NAD	9/9	7/7	1/3	6/10	1/7	1/7		5/19				3/17	1/10		2/10				
Raised scales and loss							1/6	1/19	1/7			1/17	1/10						
Epithelium attenuation and loss			1/3	2/10	3/7	5/7	2/6	3/19	5/7	7/7		3/17	5/10			3/5		3/4	1/5
Sloughing				1/10	4/7	4/7	2/6	8/19	4/7	3/7	1/4	5/17	3/10	3/4	7/10		1/2	2/4	2/5
Epithelial erosion				1/10	2/7	3/7	3/6	7/19	3/7	2/7	4/4	3/17		3/4	5/10	2/5			
Scale pocket oedema							1/6			3/7		2/17	1/10				1/2		
Inflammatory cells in the epidermis								1/19				1/17			2/10				
Focal dermatitis			1/3				3/6	4/19	3/7	2/7		3/17			2/10	2/5		2/4	4/5
Dermal haemorrhaging							1/6	1/19					1/10			2/5			
Necrosis								1/19											
Epithelial hyperplasia							1/6	4/19			2/4	6/17			3/10				1/5
Epithelial oedema					1/7		1/6	2/19	2/7	4/7	2/4	2/17	4/10	4/4	2/10	1/5	2/2	3/4	
Rodlet Cells															1/10				
Scale regeneration								1/19							1/10				
RMS												1/17							1/5
*Ichthyobodo necator*																		2/4	
*Ichthyophthirius multifiliis*							1/6		1/7	1/7				1/4				1/4	

dpc: days post challenge. NAD: no abnormalities detected. TA: Trojan from site A; TB: Trojan from site B.

The parasite *I*. *multifiliis* was observed in challenged fish and in Trojans (both also presenting with clinical signs of PSD), but not in the control fish. Epithelial hyperplasia and oedema were also observed in the gills of challenged fish. *I*. *necator* and the microsporidian *Loma salmonae* was observed in Trojans (S1 Table). RMS-like lesions were observed at the end of the challenge in one naïve fish cohabited with Trojan fish from site B, as well as in one of the Trojan fish from that site. Histologically, dermal lymphocytic infiltration typical of RMS [4] was confirmed in both fish. In internal organs, no consistent histopathological changes were observed. Proliferative kidney disease (PKD), caused by *Tetracapsuloides bryosalmonae* associated with renal and splenic inflammation was observed in two Trojan fish from site A (S2 and S3 Tables). Mild epicarditis was observed in 2 out of 150 samples analysed (S4 Table).

### Virology and bacteriology analysis

#### Bacteriology

From Trial 1, a high number of *Acidovorax* sp. colonies (based on partial 16S rRNA gene sequence analysis) were isolated from several naïve fish showing the PS condition as well as in Trojans from skin and kidney swabs showing mixed growth (Table 3). However, these bacteria were not visualised in association with the observed pathology.

No significant bacterial isolates were obtained from naïve fish in Trial 2.

#### Virology

During Trial 1, no CPE was observed in the different fish cell lines inoculated with tissues homogenates. However, IPNV was isolated from 15% of the naïve fish that were challenged and sampled during Trial 2.

### Metagenomic sequence analysis

Sequencing of the affected and non-affected skin regions of two naïve fish from the Trial 1 resulted in the generation of 42,201,893 raw sequence reads. The number of raw reads obtained ranged from 7,939,981 to 13,866,793 and after quality trimming and removal of sequences representing rainbow trout transcripts, a total of 1,668,315 paired reads (~4%) remained (S5 Table).

Screening for small (16S/18S) and large (23S/28S) subunit rRNA genes in the full quality-trimmed paired reads dataset demonstrated the presence of a selection of eukaryotic and bacterial sequences (summarised in S7 and S8 Tables, respectively). Eukaryotic sequences identified represented a range of organisms within the phyla Annelida, Arthropoda and Echinodermata, but were dominated by members of the Craniata (a clade of the phylum Chordata in which fish belong). In addition, 18S sequences with high similarity to families of a number of known fish pathogens were also identified and included Saprolegniaceae (*Saprolegnia parasitica*), *I*. *multifiliis*, *Ophryoglena catenula and I*. *necator*). *Ichthyobodo* 18S rRNA was found in all samples, apart from the affected skin samples of fish 15 (15-A). *I*. *multifiliis* and *O*. *catenula* sequences were only found in fish 11 (both skin tissue samples), whereas *S*. *parasitica* sequences were only found in the affected skin samples of this fish (11-A). A number of different types of bacteria were identified in the various samples, including sequences of likely *Flavobacteria psychrophilum* origin [27] in samples 11-A and 15-N.

After removal of rainbow trout sequences, a total of 2636, 1775, 3281 and 3112 transcripts were assembled *de novo* for samples 11-N, 11-A, 15-N and 15-A, respectively, using the Trinity assembler (summary statistics can be found in S6 Table). Annotation of these transcripts revealed similar results to those obtained after screening for the presence of ribosomal RNA genes, with the majority of the transcripts representing host (salmonid) gene sequences. All of the fish pathogens identified above were also found in the assembled transcriptomes. *Ichthyobodo* transcripts were found across all samples (including affected and non-affected tissues), whereas transcripts representing *S*. *parasitica*, *I*. *multifiliis* and *Ophryoglena catenula* were only found in the affected and non-affected skin samples of fish 11. Sequences representing bacterial transcripts were found across the various skin samples, including for *Flavobacterium psychrophilum* (sample 11-N) and for other Flavobacteriaceae (found in samples 15-N and 15-A). Two transcripts of sample 11-N showed high homology to 23S sequences of *Rickettsia spp*. No other obvious putative fish pathogens were identified in the various skin samples. Lists of all annotated sequences (and their corresponding sequence information) for each sample are given in S1 File.

## Discussion

This is the first study showing the transmission of puffy skin disease under laboratory conditions. Naïve rainbow trout exposed by cohabitation with PS-affected fish collected from the field, developed epidermal hyperplasia and oedema and dermatitis, characteristic of PSD [2]). The disease clinical signs were first observed between 14 and 21 dpc, seemingly dependent on the source of the Trojan fish. Earlier epidemiological investigations also indicated a likely infectious aetiology for the condition [1].

For this study, the approaches used were extended from conventional bacteriology, histological or virological analyses, to also include analysis of metagenome sequence data generated from PSD samples. However, similar to the results of the earlier studies, an obvious causative agent was not identified.

In some respects, PSD shares similarities with another skin condition of rainbow trout of uncertain aetiology that has recently emerged in the UK, namely RMS [4]. A previous study [1] also showed there was a strong relationship between PSD and RMS (odds ratio (OR) of 9.7). The authors discussed that this could have been due to confounding with live fish movements, although they also noted that this association was in fact stronger than that between PSD and live fish movements (OR of 9.7 as opposed to 5.0).

Although definitive proof of the responsible agent for RMS is still lacking, there is a plausible candidate for the condition, namely a Rickettsia-like organism (RLO). Very highly similar DNA of RLO-origin has been routinely recovered from fish presenting with both what is known as strawberry disease in the US and RMS in the UK [5–7]). As a part of recent phylogenetic reconstructions of RLO’s, the partial 16S rRNA gene sequences obtained from rainbow trout affected with CWSD/RMS and deposited in GENBANK have been placed within a proposed new family, the *Midichloriaceae* [28]. This group of bacteria, phylogenetically closely related to *Midichloria midichondriae* (an intramitochondrial organism of the order Ricketsiales associated with the sheep tick *Ixodes ricinus*) are associated with a wide range of host organisms, ranging from amoebae to humans [28].

Recent work has shown that, using a quantitative PCR, [29], there was a strong relationship between lesion development in RMS-affected fish from Scotland and *Midichloria*- like organism (MLO) copy number. MLO copy numbers in lesions were higher than in other tissue samples tested. MLO was not detected in other fish either. Similar results were also reported by Metselaar, who analysed RMS-affected fish from a variety of sites, using a qPCR assay developed to detect DNA of MLO origin.

However, there are a number of factors that make a direct relationship between RMS and PSD appear unlikely. Firstly, the histological presentations of the conditions are quite distinct. Secondly, RMS emerged in the UK rainbow trout industry before PSD and is now widely observed throughout the industry. Parallel reports of fish developing PSD-like clinical signs have not been reported at the same time that RMS outbreaks were first being noted. The condition is also reportedly more prevalent in the summer and autumn, as opposed to the colder winter months more associated with RMS. Finally, the apparent incubation time of the disease, as evidenced by the first appearance of clinical signs in naïve fish exposed to cohabitant Trojan fish, is apparently much shorter for PSD than RMS. It typically takes more than 500 degree days (e.g. 50 days at 10°C) for naïve fish to develop lesions characteristic of RMS. In contrast, in this study, signs consistent with PSD were observed in naïve fish exposed to PSD-fish within 15 days of exposure at 15°C (or 225 degree days). Interestingly, naïve fish exposed to Trojan fish from site B in trial 2 did develop RMS-like clinical signs by the end of the study (49 days or 735 degree days post challenge; Table 4).

In a parallel study, we have sequenced skin samples from RMS-affected rainbow trout and sequence analysis revealed the presence of MLO-like sequences in these samples (unpublished data). Similar sequences were not recovered from any of the other samples analysed, including the PSD-fish (this study) and control and affected material from fish presenting with other skin disorders (e.g. cherry fin disease) (unpublished data). This is further evidence that, if the MLO is the causative agent of RMS, it is not the same agent that is responsible for PSD.

High infestations with *I*. *necator* can result in skin pathology similar to that observed in PSD, particularly in juvenile fish [30]. The recovery of *I*. *necator* sequences from challenged fish and observation of *I*. *necator* in approximately 10% of fresh smears analysed from Trojans in the second trial may be significant. However, caution should be taken to making an assumption that *I*. *necator* has a role in PSD. Firstly, *I*. *necator*, the causative agent of costia in trout and other freshwater fish species [31], has long been endemic within the UK trout industry. It may therefore be expected that PSD-characteristic pathology would have been noted previously. Further, although *I*. *necator* was observed in histology sections of Trojans, the relative prevalence (10% or less) and intensity of infection was low. Although this was possibly explained by removal of parasites through the process of sampling and histological processing. Furthermore, the pathogen *I*. *necator* was not directly detected in the fish sampled from the first trial, although transcripts of *I*. *necator* were recovered. However, it is possible that the pathogen itself has evolved and emergence of a more virulent variant of *I*. *necator* could be responsible for PSD.

Similarly, *I*. *multifiliis* [32] was also observed in some of the samples from PSD-fish. It was notable that during the transmission trials an early response of naive fish exposed to the PSD-fish was ‘flashing’ behaviour. It is possible that this response was induced by skin irritation initiated by infection of parasites of the skin, such as *I*. *multifilis* and *I*. *necator*. Such behaviour is typically observed in fish that are suffering skin irritation. However, as with *I*. *necator*, *I*. *multifiliis* is also endemic in the UK rainbow trout population so, again, it seems somewhat surprising that, if it did have a role in the observed pathology, that this had not been seen earlier. Particularly, as it was also observed at only a very low prevalence and intensity in sampled PSD-fish.

Rodlet cells are now regarded as highly differentiated host cells involved in the response to disease, parasitic infection or to tissue damage [33,34]. They are usually found in epithelial tissues but also in blood, connective tissues, spleen and kidney. They were observed in skin scrapes from affected fish in the present study. Although not all cells contained the characteristic ‘rodlets’ these were regarded as immature forms. In addition, some were apparently bi-nucleate, a feature which has previously been reported (Fig 10, [33]). Their presence supports the hypothesis that PSD is associated with an infective agent.

Metagenomics-based approaches have been used in a range of studies to help identify novel disease agents in clinical samples from humans and terrestrial animals, as well as plant pathology samples [35,36]. These methods have also been used to help identify pathogens of fish [37]. The inability to more definitively identify an aetiological agent using metagenomics approaches was not necessarily unexpected. The random amplification and sequencing of extracted and purified DNA, or cDNA, from tissue samples, will predominantly consist of host genome or expressed RNA material, unless the agent is of very high concentration or intensity. Identifying an agent, if it is of relatively low intensity, may well be difficult to achieve. Future studies may wish to employ additional subtractive approaches that remove a greater proportion of host-derived genetic material prior to amplification and sequencing. The risk of such approaches though, is that target pathogen-derived DNA or RNA may also be removed during these sample preparation steps. Both histology and metagenomics were however, successful in detecting potential causative agents but not in sufficient quantity to suggest that they were directly involved in the pathogenesis of PSD. Sampling a larger number of affected and unaffected fish for metagenomic analysis, and identifying statistically significant differences in the composition and abundance of micro-organisms between the two groups, may provide insights into the infectious aetiology.

In conclusion we have demonstrated that PSD is a transmissible disease. However, even though a number of known fish pathogens were identified in the skin tissues of PSD-fish, the actual causative infectious agent(s) remain(s) unknown. Further work is warranted to both try and determine the responsible agent(s) and to develop and recommend effective control strategies to minimise the impact of this emerging condition in UK farmed rainbow trout.

## Supporting Information

S1 File Lists of all annotated sequences (and their corresponding sequence information) for each sample. (XLSX)

S1 Table Histopathological changes observed in gills of rainbow trout during the second cohabitation trial with puffy skin affected fish. (DOCX)

S2 Table Histopathological changes observed in kidney of rainbow trout during the second cohabitation trial with puffy skin affected fish. (DOCX)

S3 Table Histopathological changes observed in spleen of rainbow trout during the second cohabitation trial with puffy skin affected fish. (DOCX)

S4 Table Histopathological changes observed in heart of rainbow trout during the second cohabitation trial with puffy skin affected fish. (DOCX)

S5 Table Number of reads before and after quality trimming and after removal of reads representing rainbow trout sequences. (DOCX)

S6 Table Summary statistics for the *de novo* assembled transcripts. (DOCX)

S7 Table Taxonomic classification of eukaryotic rRNA sequences in the affected and non-affected rainbow trout skin samples. (DOCX)

S8 Table Taxonomic classification of bacterial rRNA sequences in the affected and non-affected rainbow trout skin samples. (DOCX)
