# Supplementary material for: Puffy Skin Disease Is an Emerging Transmissible Condition in Rainbow Trout Oncorhynchus mykiss Walbaum
Source: PLoS One. 2016 Jul 8;11(7):e0158151. doi: 10.1371/journal.pone.0158151 (PMC4938586; doi:10.1371/journal.pone.0158151)
Supplement: S1 Table — (DOCX) [file pone.0158151.s002.docx]

**S1 Table. Histopathological changes observed in gills of rainbow trout during the second cohabitation trial with puffy skin affected fish.** dpc: days post challenge. TA: Trojan from site A; TB: Trojan from site B.

| **Gill** | **NAD** | **Epithelium lighting** | **Primary Lamellae Lesion** | **Prominent eosin cells** | **Lamellar fusion** | **Epithelium necrosis** | **Oedema** | **Hyperplasia** | **Necrosis** | **Inflammation (neutrophil)** | **LOMA** | ***Ichthyobodo necator*** | ***Ichthyophthirius multifiliis*** |
| --- | --- | --- | --- | --- | --- | --- | --- | --- | --- | --- | --- | --- | --- |
| **Negative group** |  |  |  |  |  |  |  |  |  |  |  |  |  |
| Naive fish at arrival | - |  |  |  |  |  |  |  |  |  |  |  |  |
| Control fish at 2 wpc^1^ | - |  |  |  |  |  |  |  |  |  |  |  |  |
| Control fish at 3 wpc | - |  |  |  |  |  |  |  |  |  |  |  |  |
| Control fish at 7 wpc | 8/10 | 2/10 |  |  |  |  |  |  |  |  |  |  |  |
| **Naive fish in cohabitation with Trojan A** |  |  |  |  |  |  |  |  |  |  |  |  |  |
| 2 wpc | - |  |  |  |  |  |  |  |  |  |  |  |  |
| 3 wpc | - |  |  |  |  |  |  |  |  |  |  |  |  |
| 5 wpc |  | 3/6 |  |  | 1/6 |  | 1/6 | 4/6 |  |  |  |  |  |
| 7 wpc | 13/19 | 2/19 |  | 6/19 | 4/19 | 4/19 | 1/19 | 2/19 |  |  |  |  |  |
| **Naive fish in cohabitation with Trojan B** |  |  |  |  |  |  |  |  |  |  |  |  |  |
| 2 wpc | - |  |  |  |  |  |  |  |  |  |  |  |  |
| 3 wpc | - |  |  |  |  |  |  |  |  |  |  |  |  |
| 5 wpc | 2/4 |  |  |  | 1/4 |  |  |  | 1/4 |  |  |  | 1/4 |
| 7 wpc | 8/17 | 6/17 |  | 1/17 |  |  |  | 1/17 |  |  |  |  |  |
| **Trojan A** |  |  |  |  |  |  |  |  |  |  |  |  |  |
| Trojan A at arrival |  | 1/1 |  |  | 1/1 |  |  | 1/1 |  |  |  |  |  |
| 5 wpc | 1/4 | 3/4 |  | 1/4 |  |  | 3/4 | 2/4 |  |  | 1/4 |  | 2/4 |
| 7 wpc | 9/10 |  | 1/10 |  |  |  |  |  |  |  |  |  |  |
| **Trojan B** |  |  |  |  |  |  |  |  |  |  |  |  |  |
| Trojan B at arrival | - |  |  |  |  |  |  |  |  |  |  |  |  |
| 3 wpc | - |  |  |  |  |  |  |  |  |  |  |  |  |
| 5 wpc | 1/4 | 4/4 |  |  | 2/4 |  | 2/4 | 2/4 |  | 1/4 |  | 1/4 | 1/4 |
| 7 wpc | 2/5 |  |  | 1/5 | 1/5 |  |  | 1/5 |  |  |  |  |  |
