# Supplementary material for: Puffy Skin Disease Is an Emerging Transmissible Condition in Rainbow Trout Oncorhynchus mykiss Walbaum
Source: PLoS One. 2016 Jul 8;11(7):e0158151. doi: 10.1371/journal.pone.0158151 (PMC4938586; doi:10.1371/journal.pone.0158151)
Supplement: S3 Table — (DOCX) [file pone.0158151.s004.docx]

**S3 Table. Histopathological changes observed in spleen of rainbow trout during the second cohabitation trial with puffy skin affected fish.** dpc: days post challenge. TA: Trojan from site A; TB: Trojan from site B.

| **Spleen** | **NAD** | **Congestion** | **Necrosis** | **Increased macrophage MA** | **PKD** | **Splenomegaly** | **Cell granulation** |
| --- | --- | --- | --- | --- | --- | --- | --- |
| **Negative group** |  |  |  |  |  |  |  |
| Naive fish at arrival | 9/10 | 1 |  |  |  |  |  |
| Control fish at 2 wpc^1^ | 7/7 |  |  |  |  |  |  |
| Control fish at 3 wpc | 3/3 |  |  |  |  |  |  |
| Control fish at 7 wpc | 7/10 | 3 |  |  |  |  |  |
| **Naive fish in cohabitation with Trojan A** |  |  |  |  |  |  |  |
| 2 wpc | 7/7 |  |  |  |  |  |  |
| 3 wpc | 7/7 |  |  |  |  |  |  |
| 5 wpc | 6/6 |  |  |  |  |  |  |
| 7 wpc | 15/19 | 1 |  | 2 | 2 | 1 | 1 |
| **Naive fish in cohabitation with Trojan B** |  |  |  |  |  |  |  |
| 2 wpc | 7/7 |  |  |  |  |  |  |
| 3 wpc | 7/7 |  |  |  |  |  |  |
| 5 wpc | 4/4 |  |  |  |  |  |  |
| 7 wpc | 16/17 | 1 | 1 |  |  |  |  |
| **Trojan A** |  |  |  |  |  |  |  |
| Trojan A at arrival | 8/10 |  |  |  |  | 2 |  |
| 5 wpc | 4/4 |  |  |  |  |  |  |
| 7 wpc | 10/10 |  |  |  |  |  |  |
| **Trojan B** |  |  |  |  |  |  |  |
| Trojan B at arrival | 3/5 |  |  |  |  |  |  |
| 3 wpc | 2/2 |  |  |  |  |  |  |
| 5 wpc | 4/4 |  |  |  |  |  |  |
| 7 wpc | 5/5 |  |  |  |  |  |  |
