# Supplementary material for: Puffy Skin Disease Is an Emerging Transmissible Condition in Rainbow Trout Oncorhynchus mykiss Walbaum
Source: PLoS One. 2016 Jul 8;11(7):e0158151. doi: 10.1371/journal.pone.0158151 (PMC4938586; doi:10.1371/journal.pone.0158151)
Supplement: S5 Table — (DOCX) [file pone.0158151.s006.docx]

**S5 Table. Number of reads before and after quality trimming and after removal of reads representing rainbow trout sequences.**

| **Sample** | **Raw Reads** | **Trimmed paired reads** | **Trimmed orphan reads (F)** | **Trimmed orphan reads (R)** | **Trimmed paired reads after removal of rainbow trout sequences** |
| --- | --- | --- | --- | --- | --- |
| 11-PS-N | 13,866,793 | 13,416,310 | 343,407 | 37,090 | 610,653 |
| 11-PS-A | 7,939,981 | 7,690,236 | 203,762 | 26,690 | 294,097 |
| 15-PS-N | 9,561,438 | 9,377,898 | 153,429 | 20,776 | 333,520 |
| 15-PS-A | 11,935,813 | 11,717,449 | 178,372 | 19,111 | 430,045 |
