# Supplementary material for: Puffy Skin Disease Is an Emerging Transmissible Condition in Rainbow Trout Oncorhynchus mykiss Walbaum
Source: PLoS One. 2016 Jul 8;11(7):e0158151. doi: 10.1371/journal.pone.0158151 (PMC4938586; doi:10.1371/journal.pone.0158151)
Supplement: S6 Table — (DOCX) [file pone.0158151.s007.docx]

**S6 Table. Summary statistics for the *de novo* assembled transcripts**

| **Sample** | **No of Transcripts** | **Total Size (bp)** | **Min Size (bp)** | **Max Size (bp)** | **Average Size (bp)** | **Median Size (bp)** | **N50** |
| --- | --- | --- | --- | --- | --- | --- | --- |
| 11-PS-N | 2,636 | 872,477 | 201 | 4,982 | 330.99 | 253 | 306 |
| 11-PS-A | 1,775 | 621,529 | 201 | 4,269 | 350.16 | 258 | 326 |
| 15-PS-N | 3,281 | 1,213,957 | 201 | 6,018 | 370 | 266 | 353 |
| 15-PS-A | 3,112 | 1,056,499 | 201 | 4,090 | 339.49 | 258 | 315 |
