# Supplementary material for: Puffy Skin Disease Is an Emerging Transmissible Condition in Rainbow Trout Oncorhynchus mykiss Walbaum
Source: PLoS One. 2016 Jul 8;11(7):e0158151. doi: 10.1371/journal.pone.0158151 (PMC4938586; doi:10.1371/journal.pone.0158151)
Supplement: S7 Table — (DOCX) [file pone.0158151.s008.docx]

**S7 Table. Taxonomic classification of eukaryotic rRNA sequences in the affected and non-affected rainbow trout skin samples.** Normalised reads are presented as counts per 10 million reads.

|  | **Number of reads per taxonomic group** | | | | **Normalised reads (counts per 10 million)** | | | |
| --- | --- | --- | --- | --- | --- | --- | --- | --- |
| **Taxonomy** | 11_PS_N | 11_PS_A | 15_PS_N | 15_PS_A | 11_PS_N | 11_PS_A | 15_PS_N | 15_PS_A |
| Eukaryota;Alveolata;Ciliophora;Intramacronucleata;Conthreep | 2 | 43 | 0 | 0 | 1.5 | 55.9 | 0.0 | 0.0 |
| Eukaryota;Chromalveolata;Stramenopiles;  Peronosporomycetes;Saprolegnia | 0 | 1 | 0 | 0 | 0.0 | 1.3 | 0.0 | 0.0 |
| Eukaryota;Chromalveolata;Stramenopiles;  Peronosporomycetes;Unclassified Peronosporomycetes | 0 | 3 | 0 | 0 | 0.0 | 3.9 | 0.0 | 0.0 |
| Eukaryota;Excavata;Euglenozoa;Kinetoplastea;  Prokinetoplastina | 2 | 31 | 3 | 0 | 1.5 | 40.3 | 3.2 | 0.0 |
| Eukaryota;Fungi;Ascomycota;Saccharomycotina;  Saccharomycetales | 0 | 0 | 0 | 1 | 0.0 | 0.0 | 0.0 | 0.9 |
| Eukaryota;Metazoa;Annelida;Annelida;Family Incertae Sedis | 23 | 247 | 341 | 42 | 17.1 | 321.2 | 363.6 | 35.8 |
| Eukaryota;Metazoa;Annelida;Annelida;Unclassified Annelida | 0 | 0 | 7 | 2 | 0.0 | 0.0 | 7.5 | 1.7 |
| Eukaryota;Metazoa;Annelida;Unclassified Annelida; | 1 | 38 | 62 | 12 | 0.7 | 49.4 | 66.1 | 10.2 |
| Eukaryota;Metazoa;Arthropoda;Chelicerata;Arachnida | 0 | 3 | 2 | 0 | 0.0 | 3.9 | 2.1 | 0.0 |
| Eukaryota;Metazoa;Arthropoda;Crustacea;Maxillopoda | 0 | 0 | 1 | 0 | 0.0 | 0.0 | 1.1 | 0.0 |
| Eukaryota;Metazoa;Arthropoda;Crustacea;Unclassified Crustacea | 0 | 0 | 1 | 0 | 0.0 | 0.0 | 1.1 | 0.0 |
| Eukaryota;Metazoa;Arthropoda;Hexapoda;Collembola | 0 | 1 | 0 | 0 | 0.0 | 1.3 | 0.0 | 0.0 |
| Eukaryota;Metazoa;Arthropoda;Hexapoda;Insecta | 16 | 291 | 534 | 68 | 11.9 | 378.4 | 569.4 | 58.0 |
| Eukaryota;Metazoa;Arthropoda;Hexapoda;Unclassified Hexapoda | 0 | 4 | 4 | 0 | 0.0 | 5.2 | 4.3 | 0.0 |
| Eukaryota;Metazoa;Arthropoda;Unclassified Arthropoda; | 0 | 36 | 33 | 1 | 0.0 | 46.8 | 35.2 | 0.9 |
| Eukaryota;Metazoa;Ascidiacea;Unclassified Ascidiacea; | 0 | 1 | 1 | 0 | 0.0 | 1.3 | 1.1 | 0.0 |
| Eukaryota;Metazoa;Bryozoa;Bryozoa;Family Incertae Sedis | 0 | 0 | 1 | 0 | 0.0 | 0.0 | 1.1 | 0.0 |
| Eukaryota;Metazoa;Cephalochordata;Unclassified Cephalochordata; | 0 | 0 | 1 | 0 | 0.0 | 0.0 | 1.1 | 0.0 |
| Eukaryota;Metazoa;Chordata;Craniata;Craniata | 4393 | 153528 | 513754 | 40665 | 3274.4 | 199640.2 | 547834.9 | 34704.7 |
| Eukaryota;Metazoa;Chordata;Craniata;Unclassified Craniata | 0 | 17 | 39 | 3 | 0.0 | 22.1 | 41.6 | 2.6 |
| Eukaryota;Metazoa;Chordata;Unclassified Chordata; | 1 | 204 | 240 | 6 | 0.7 | 265.3 | 255.9 | 5.1 |
| Eukaryota;Metazoa;Cnidaria;Cnidaria;Anthozoa | 0 | 1 | 4 | 0 | 0.0 | 1.3 | 4.3 | 0.0 |
| Eukaryota;Metazoa;Echinodermata;Echinodermata;  Family Incertae Sedis | 4 | 48 | 101 | 9 | 3.0 | 62.4 | 107.7 | 7.7 |
| Eukaryota;Metazoa;Entoprocta;Entoprocta;Pedicellinidae | 0 | 1 | 2 | 2 | 0.0 | 1.3 | 2.1 | 1.7 |
| Eukaryota;Metazoa;Mollusca;Bivalvia;Anomalodesmata | 0 | 0 | 1 | 0 | 0.0 | 0.0 | 1.1 | 0.0 |
| Eukaryota;Metazoa;Mollusca;Bivalvia;Heteroconchia | 0 | 1 | 3 | 0 | 0.0 | 1.3 | 3.2 | 0.0 |
| Eukaryota;Metazoa;Mollusca;Gastropoda;Caenogastropoda | 0 | 0 | 1 | 0 | 0.0 | 0.0 | 1.1 | 0.0 |
| Eukaryota;Metazoa;Mollusca;Gastropoda;Unclassified Gastropoda | 0 | 1 | 0 | 0 | 0.0 | 1.3 | 0.0 | 0.0 |
| Eukaryota;Metazoa;Mollusca;Unclassified Mollusca; | 0 | 0 | 1 | 0 | 0.0 | 0.0 | 1.1 | 0.0 |
| Eukaryota;Metazoa;Nematoda;Chromadorea;Unclassified Chromadorea | 0 | 1 | 0 | 0 | 0.0 | 1.3 | 0.0 | 0.0 |
| Eukaryota;Metazoa;Nematoda;Enoplea;Mermithidae | 0 | 0 | 1 | 0 | 0.0 | 0.0 | 1.1 | 0.0 |
| Eukaryota;Metazoa;Nematoda;Enoplea;Unclassified Enoplea | 0 | 1 | 0 | 0 | 0.0 | 1.3 | 0.0 | 0.0 |
| Eukaryota;Metazoa;Platyhelminthes;Cestoda;Eucestoda | 0 | 0 | 1 | 0 | 0.0 | 0.0 | 1.1 | 0.0 |
| Eukaryota;Metazoa;Platyhelminthes;Monogenea;  Monopisthocotylea | 0 | 0 | 1 | 0 | 0.0 | 0.0 | 1.1 | 0.0 |
| Eukaryota;Metazoa;Platyhelminthes;Trematoda;Digenea | 0 | 1 | 1 | 0 | 0.0 | 1.3 | 1.1 | 0.0 |
| Eukaryota;Metazoa;Platyhelminthes;Turbellaria;Seriata | 1 | 49 | 41 | 7 | 0.7 | 63.7 | 43.7 | 6.0 |
| Eukaryota;Metazoa;Platyhelminthes;Turbellaria;Unclassified Turbellaria | 0 | 1 | 3 | 0 | 0.0 | 1.3 | 3.2 | 0.0 |
| Eukaryota;Metazoa;Platyhelminthes;Unclassified Platyhelminthes; | 0 | 0 | 5 | 0 | 0.0 | 0.0 | 5.3 | 0.0 |
| Eukaryota;Metazoa;Unclassified Metazoa;; | 368 | 11091 | 30886 | 2759 | 274.3 | 14422.2 | 32934.9 | 2354.6 |
| Eukaryota;Unclassified Eukaryota;;; | 3 | 30 | 65 | 2 | 2.2 | 39.0 | 69.3 | 1.7 |
| Eukaryota;Viridiplantae;Streptophyta;Embryophyta;Coniferales | 0 | 1 | 0 | 0 | 0.0 | 1.3 | 0.0 | 0.0 |
| Eukaryota;Viridiplantae;Streptophyta;Embryophyta;  Unclassified Embryophyta | 0 | 1 | 1 | 0 | 0.0 | 1.3 | 1.1 | 0.0 |
