# Supplementary material for: Puffy Skin Disease Is an Emerging Transmissible Condition in Rainbow Trout Oncorhynchus mykiss Walbaum
Source: PLoS One. 2016 Jul 8;11(7):e0158151. doi: 10.1371/journal.pone.0158151 (PMC4938586; doi:10.1371/journal.pone.0158151)
Supplement: S8 Table — (DOCX) [file pone.0158151.s009.docx]

**S8 Table. Taxonomic classification of bacterial rRNA sequences in the affected and non-affected rainbow trout skin samples.** Normalised reads are presented as counts per 10 million reads.

|  | **Number of reads per taxonomic group** | | | | **Normalised reads (counts per 10 million)** | | | |
| --- | --- | --- | --- | --- | --- | --- | --- | --- |
| **Taxonomy** | 11_PS_N | 11_PS_A | 15_PS_N | 15_PS_A | 11_PS_N | 11_PS_A | 15_PS_N | 15_PS_A |
| Bacteria;Bacteroidetes;Cytophagia;Cytophagales;  Cytophagaceae | 0 | 0 | 1 | 0 | 0.0 | 0.0 | 1.1 | 0.0 |
| Bacteria;Bacteroidetes;Flavobacteria;  Flavobacteriales;Flavobacteriaceae | 0 | 1 | 7 | 0 | 0.0 | 1.3 | 7.5 | 0.0 |
| Bacteria;Bacteroidetes;Sphingobacteriia;  Sphingobacteriales;Sphingobacteriaceae | 0 | 0 | 1 | 0 | 0.0 | 0.0 | 1.1 | 0.0 |
| Bacteria;Firmicutes;Bacilli;Lactobacillales;  Streptococcaceae | 0 | 0 | 1 | 0 | 0.0 | 0.0 | 1.1 | 0.0 |
| Bacteria;Firmicutes;Clostridia;Clostridiales;  Veillonellaceae | 1 | 0 | 0 | 0 | 0.7 | 0.0 | 0.0 | 0.0 |
| Bacteria;Fusobacteria;Fusobacteria;Fusobacteriales;  Leptotrichiaceae | 0 | 0 | 1 | 0 | 0.0 | 0.0 | 1.1 | 0.0 |
| Bacteria;Proteobacteria;Alphaproteobacteria;  Caulobacterales;Caulobacteraceae | 0 | 0 | 7 | 1 | 0.0 | 0.0 | 7.5 | 0.9 |
| Bacteria;Proteobacteria;Alphaproteobacteria;  Sphingomonadales;Sphingomonadaceae | 0 | 0 | 1 | 0 | 0.0 | 0.0 | 1.1 | 0.0 |
| Bacteria;Proteobacteria;Betaproteobacteria;  Burkholderiales;Comamonadaceae | 0 | 1 | 1 | 0 | 0.0 | 1.3 | 1.1 | 0.0 |
| Bacteria;Proteobacteria;Betaproteobacteria;  Burkholderiales;Oxalobacteraceae | 0 | 0 | 1 | 0 | 0.0 | 0.0 | 1.1 | 0.0 |
| Bacteria;Proteobacteria;Gammaproteobacteria;  Pseudomonadales;Moraxellaceae | 0 | 0 | 7 | 0 | 0.0 | 0.0 | 7.5 | 0.0 |
| Bacteria;Proteobacteria;Gammaproteobacteria;  Pseudomonadales;Pseudomonadaceae | 0 | 0 | 1 | 0 | 0.0 | 0.0 | 1.1 | 0.0 |
| Bacteria;Proteobacteria;Gammaproteobacteria;  Xanthomonadales;Xanthomonadaceae | 0 | 0 | 2 | 0 | 0.0 | 0.0 | 2.1 | 0.0 |
